# Allelochemical run-off from the invasive terrestrial plant *Impatiens glandulifera* decreases defensibility in *Daphnia*

**DOI:** 10.1038/s41598-023-27667-4

**Published:** 2023-01-21

**Authors:** Jens Georg Peter Diller, Frederic Hüftlein, Darleen Lücker, Heike Feldhaar, Christian Laforsch

**Affiliations:** 1 Animal Ecology I, Universitaetsstraße 30, 95447 Bayreuth, Germany; 2 BayCEER, Universitaetsstraße 30, 95447 Bayreuth, Germany

**Keywords:** Freshwater ecology, Invasive species, Limnology, Environmental impact

## Abstract

Invasive species are a major threat for native ecosystems and organisms living within. They are reducing the biodiversity in invaded ecosystems, by outcompeting native species with e. g. novel substances. Invasive terrestrial plants can release allelochemicals, thereby reducing biodiversity due to the suppression of growth of native plants in invaded habitats. Aside from negative effects on plants, allelochemicals can affect other organisms such as mycorrhiza fungi and invertebrates in terrestrial ecosystems. When invasive plants grow in riparian zones, it is very likely that terrestrial borne allelochemicals can leach into the aquatic ecosystem. There, the often highly reactive compounds may not only elicit toxic effects to aquatic organisms, but they may also interfere with biotic interactions. Here we show that the allelochemical 2-methoxy-1,4-naphthoquinone (2-MNQ), produced by the ubiquitously occurring invasive terrestrial plant *Impatiens glandulifera*, interferes with the ability of *Daphnia* to defend itself against predators with morphological defences. *Daphnia magna* and *Daphnia longicephala* responded with morphological defences induced by chemical cues released by their corresponding predators, *Triops cancriformis* or *Notonecta sp*. However, predator cues in combination with 2-MNQ led to a reduction in the morphological defensive traits, body- and tail-spine length, in *D. magna*. *In D. longicephala* all tested inducible defensive traits were not significantly affected by 2-MNQ but indicate similar patterns, highlighting the importance to study different species to assess the risks for aquatic ecosystems. Since it is essential for *Daphnia* to adapt defences to the current predation risk, a maladaptation in defensive traits when simultaneously exposed to allelochemicals released by *I. glandulifera*, may therefore have knock-on effects on population dynamics across multiple trophic levels, as *Daphnia* is a key species in lentic ecosystems.

## Introduction

Interspecific interactions, such as predator–prey interactions, are a main driver for shaping the composition of a community^1^. There, both species need to invest energy, the predator in searching and hunting, the prey in escape or development of defences against the weaponry of the predator^2,3^.

Due to the selection pressure exerted by various predators, prey species have evolved different defence strategies: Constitutive and inducible defences. Conspicuous examples of constitutive defences are for instance the quills of a hedgehog or the shell of a turtle^4,5^. In contrast, other prey species develop inducible defences, which are predator specific and are only expressed when the corresponding predator appears in the system^6^. The risk of predation can be assessed from the prey by the detection of so called kairomones, chemical cues released by the predator which are beneficial to the receiver but not to the sender^7^. For example, mud crabs, *Panopeus herbstii*, detect the urinary metabolites trigonelline and homarine released by their crustacean predators blue crabs, *Callinectus sapidus*. In response to these chemical cues foraging by mud crabs decreases to avoid encounter with their predators^8^. Other well studied examples of inducible defences can be found in freshwater crustaceans in the order of Cladocera^9^. They respond to the presence of predators with behavioural changes such as an altered diel vertical migration or life-history changes such as an altered age at maturity^10,11^. However, the most conspicuous inducible defences are changes in morphological traits^12^: Juveniles of *Daphnia longispina* or *D. pulex*, for example, form neckteeth when exposed to the larvae of the phantom midge, *Chaoborus *sp.^13,14^*.* Other *Daphnia* species form large helmets^15^, a crown of thorns^16^ or elongated spines^17^ in response to their predators. *D. longicephala* forms a so called crest, an enormous alteration of its head shape, and increases its tail-spine length when exposed to notonectids^18^. A well studied model system in environmental research is *D. magna* which is known to express morphological defences such as an increased tail-spine length or an increased bulkiness in response to the tadpole shrimp *Triops cancriformis*^19^. Further, daphnids not only adjust their defences to the predator type^20,21^ but also to predator and conspecific density and therefore fine-tune their defences in response to the actual predation risk^22^.

It is already known that the induction of defensive traits can be affected by anthropogenic stressors. For instance rising CO_2_ levels reduce the ability of *Daphnia* to sense their predators, which will reduce the expression of inducible defences^23^*.* It was further shown that chemical compounds such as pesticides can alter defensive traits in *Daphnia*^11,24,25^. For instance, the insecticide carbaryl limits the growth of crest size of predator-exposed *D. longicephala*^11^. These chemicals used in agriculture enter aquatic ecosystems mainly via runoff from terrestrial ecosystems during precipitation^26^.

Runoff from terrestrial systems adjacent to water bodies can further contain allelochemicals, released by terrestrial plants^27,28^. Allelochemicals are produced by plants to cope with herbivores or to inhibit the germination and growth of competitors^29^. When allelochemical producing plants grow in low densities, the amount of allelochemicals leaching into adjacent waters may be negligible. However, it was shown for example that phytotoxins, such as pyrrolizidine alkaloids (PA), produced by *Senecio jacobaea* or *Petasites hybridus*, were found in concentrations up to 90 ng/l in small streams and up to 230 ng/l in seepage water from groundwater wells^28,30^. Furthermore, rainfall increased PA concentrations by a factor of ten in stream water, which might be critical for aquatic ecosystems during rainy season^28^. As a consequence, with increased abundance along the shoreline of rivers or ponds, the amount of leaching allelochemicals introduced into the water body should also increase^28^.

Hence, leachates from invasive plants, which often form monospecific stands in riparian zones, may have even wider implications for native aquatic organisms, as they probably release larger amounts of allelochemicals into the water due to their sheer number. Native aquatic species are unaccustomed to these novel toxic compounds and therefore are often not adapted to them^31^. One example for an invasive plant species that establishes monospecific stands facilitated by the production of allelochemicals and therefore reducing biodiversity of native plants is *Impatiens glandulifera*, the Himalayan Balsam. Introduced to Europe in the early nineteenth century, the plant is nowadays widespread along rivers and ponds in the northern hemisphere^32^ and has spread in parts of the southern hemisphere as well, e. g. in New Zealand^33^. First studies have shown that one allelopathic substance, 2-methoxy-1,4-naphthoquinone (2-MNQ), leaches out from leaves and can reach concentrations up to 12 mg/l in rainfall runoff^27,34^. 2-MNQ affects the growth and development of competing plants, e. g. *Urtica dioica*, by reducing for example the shoot and root growth^34–36^.

1-4-naphthoquionones, to which 2-MNQ belongs, are a class of redox-active molecules that may cause oxidative stress in cells and have been shown to increase the mortality in parasites (*Trypanosoma brucei*), human cancer cells, or aquatic invertebrates (*Eurytemora affinis*)^37–39^. In sub-lethal concentrations such compounds can affect the fitness of organisms either by reducing growth, reducing fecundity or compromising the immune system^30,40^. It was shown that a similar compound, juglone (5-hydroxy-1,4-naphthoquinone), an allelochemical produced by the black walnut tree, *Juglans nigra*, can increase the mortality in *D. magna*, when the animals are treated with finely ground walnut hulls or the purified substance^37,41^. Recently, for 2-MNQ below 12 mg/l it has been shown to alter fitness of *D. magna* as well by increasing the mortality (EC_50_ = 2.84 mg/l), reducing body size (EC_50_ = 0.649 mg/l) and number offspring (EC_50_ = 1.60 mg/l)^42^. However, if allelochemicals released by terrestrial invasive plants do interfere with the expression of chemically induced inducible defensive traits in different *Daphnia* species, keystone species in lakes and ponds, is not studied yet.

We hypothesized that non-lethal concentrations of the allelochemical 2-MNQ, originating from the invasive terrestrial plant *I. glandulifera* limit the expression of inducible morphological defences in *D. magna* and *D. longicephala*. The predator–prey systems were chosen, because inducible morphological defensive traits of both *Daphnia* species to the respective predator are well described and pronounced, ensuring that alterations in the expression of these defences in response to allelochemicals released by *I. glandulifera* can be detected.

## Material and methods

### Animal husbandry

We confirm that all experiments were performed with relevant guidelines and regulations.

### *Daphnia* stock culture

The clone of *D. longicephala* was originally isolated from a pond in Lara (38°01′38.5′′ S 144°23′58.5′′ E), Australia in 1998. The *D. magna* (K34J) clone was isolated from a former fishpond near Munich, Germany (48°12′25.3′′ N 11°43′14.5′′ E) in 1998.

The animals where cultured in a climate chamber with constant temperature at 20 ± 0.1 °C, and a 14 h: 9 h light: dark cycle with 30 min dusk and dawn. The used culture medium was a slightly modified M4-medium, where additional SeO_2_ (4.48 µg/l) was added^43^. The stock culture medium was exchanged every two days to prevent an accumulation of exuviae and algal remains. Age synchronised animals (< 24 h) were used for cultivation and were held in a cohort of less than 15 adult individuals per litre in 1.5 l glass-beakers (Weck GmbH u. Co. KG, Wehr-Öflingen, Germany), to avoid crowding effects. The daphnids were fed daily ad libitum with the green algae *Acutodesmus obliquus* and mothers were immediately separated from their offspring. The third brood of the original mother individuals were chosen to perform the experiments, since the third brood is known to be more homogenous than the first two broods^44^.

### Predator stock culture

A laboratory cultured clonal line of *T. cancriformis* originally provided by Erich Eder (Department of Evolutionary Anthropology, University of Vienna) was used for the experiments. The animals in the stock culture were fed daily with two pellets of JBL Grana (JBL GmbH & Co. KG, Neuhofen) and JBL Grana Discus (JBL GmbH & Co. KG, Neuhofen) each. They were kept at a 12 h: 12 h light: dark cycle and at a temperature of 20 ± 0.1 °C.

The clone of *D. longicephala* used in this study is known to express inducible defences against the European backswimmer (*Notonecta* sp.), which is similar to the Australian backswimmer^45^. Eggs of *Notonecta* sp. were collected from artificial ponds outside the building of the Natural Sciences I of the University of Bayreuth (49°55′44.2′′ N 11°35′00.1′′ E). The eggs were hatched in the laboratory in 12 l aquaria with M4-medium at the same light and temperature conditions (20 ± 0.1 °C; 14 h : 9 h light : dark cycle and 30 min dusk and dawn) as both *Daphnia* species. The animals in the stock culture were fed daily with a mixture of *D. longicephala* and *D. magna* individuals ad libitum.

### Chemicals

We comply with the IUCN Policy Statement on Research Involving Species at Risk of Extinction and the Convention on the Trade in Endangered Species of Wild Fauna and Flora. The source for *Impatiens glandulifera* is a leave isolate, which we purchased from Merck KGaA: https://www.sigmaaldrich.com/DE/de/product/aldrich/189162, as purified 2-methoxy-1,4-naphthoquione (2-MNQ) (Merck KGaA, CAS-number: 2348-82-5, Darmstadt, Germany; 98% purity isolated from *I. glandulifera* leaves). Due to its low solubility in water, the 2-MNQ powder was dissolved in 100 µl DMSO (Dimethylsulfoxide 99.7% purity; Bernd Kraft GmbH, Duisburg, Germany) per litre medium^42^.

### Kairomone preparation

To obtain kairomone conditioned medium, two individuals of *T. cancriformis* or *Notonecta sp.* were held in 1.5 l beakers in 1 l M4-medium for 24 h and solely fed them with individuals of *D. longicephala* or *D. magna*, respectively, to also include so called alarm cues, unintentionally released by wounded conspecifics, in the kairomone conditions water^46^. This ensures an increase in the expression of the defensive trait. Predators were held in a climate chamber with constant conditions of 20 ± 0.1 °C and a 14 h: 9 h light: dark cycle and 30 min dusk and dawn. To remove faeces and food residues, the kairomone conditioned medium was filtered through a filter paper (Whatman® filter paper Grade 4, cellulose filters, diameter 110 mm, Freiburg, Germany) with a water jet vacuum pump (suction capacity: 400 l/h; Brand GmbH & Co. KG, Wertheim, Germany)^45^. The control medium was treated the same way (with *D. longicephala* or *D. magna* individuals for 24 h) to ensure comparability.

### Effects of 2-MNQ on the expression of induced defences

We compared the effect of 2-MNQ on the expression of inducible defences in both *Daphnia* species (2-MNQ & kairomones) to animals that were solely exposed to kairomones of predators and to control animals not exposed to any of these compounds^42^. We performed acute toxicity studies in Diller et al.^42^. We determined an EC50 of 2.84 mg/l (95%—Confidence interval: 2.809 and 2.869 mg/l). Based on these results, we selected two sub-lethal 2-MNQ concentrations (0.75 mg/l and 1.5 mg/l). Individual neonates (younger than 24 h) were randomly placed into 160 ml beakers containing either 100 ml M4-medium (control), 100 ml M4-medium with either DMSO only (solvent control), 2-MNQ and DMSO, kairomone medium, kairomone medium with either DMSO only, or kairomone medium with 2-MNQ and DMSO (n = 20 per treatment). Additionally, 2 mgC/l (mgC = milligram Carbon) of the alga *A. obliquus* was added for food supply daily. *D. magna* were exposed to kairomones from *T. cancriformis* and *D. longicephala* to kairomones from *Nototecta* sp. The experiment was terminated after the last animal reached maturity and every animal was conserved in 80% ethanol for further analysis.

### Morphological measurements

For the morphological measurements, the animals were photographed under a dissecting microscope equipped with a digital camera (Leica M50, Wetzlar, Germany; camera: OLYMPUS DP26, Hamburg, Germany, Light: Leica KL 300 LED, Wetzlar, Germany) and analysed using the software cellSens Dimension (v1.11, OLYMPUS, Hamburg, Germany). For both species different parameters were defined following the methods of Rabus and Laforsch^19^ and Trotter et al.^45^ (Supplemental Data: Fig. S1): the body length was measured from the base of the tail-spine to the anterior end of the compound eye. The crest-width for *D. longicephala* was measured from the left margin of the compound eye to the right lateral margin of the head. The tail-spine length for *D. magna* was measured from the base to its tip. Unlike the straight tail spine of *D. magna,* the tail spine of *D. longicephala* grows in a curve, and therefore a polygonal line along the tail-spine was used. The crest-height was calculated by subtracting the body length from the total length.

### Statistical analysis

Statistical analysis was performed using SPSS (IBM SPSS Statistics Version 21, 64-Bit Edition, IBM Deutschland GmbH, Ehningen, Deutschland). To determine if the data was normally distributed, a Shapiro–Wilk-test was performed. To test for homogeneity of variances, a Levene-Test was performed. When the assumptions of normal distribution and homogeneity of variances were met, an analysis of variances (ANOVA) followed by a post-hoc test (Tukey-HSD) was performed. When the assumptions were not met, a non-parametric Kruskal–Wallis-Test paired with a Dunn-Bonferroni post-hoc test was performed.

## Results

### Effects of 2-MNQ on morphological parameters of kairomone-exposed *D. magna* and *D. longicephala*

**Figure 1:**
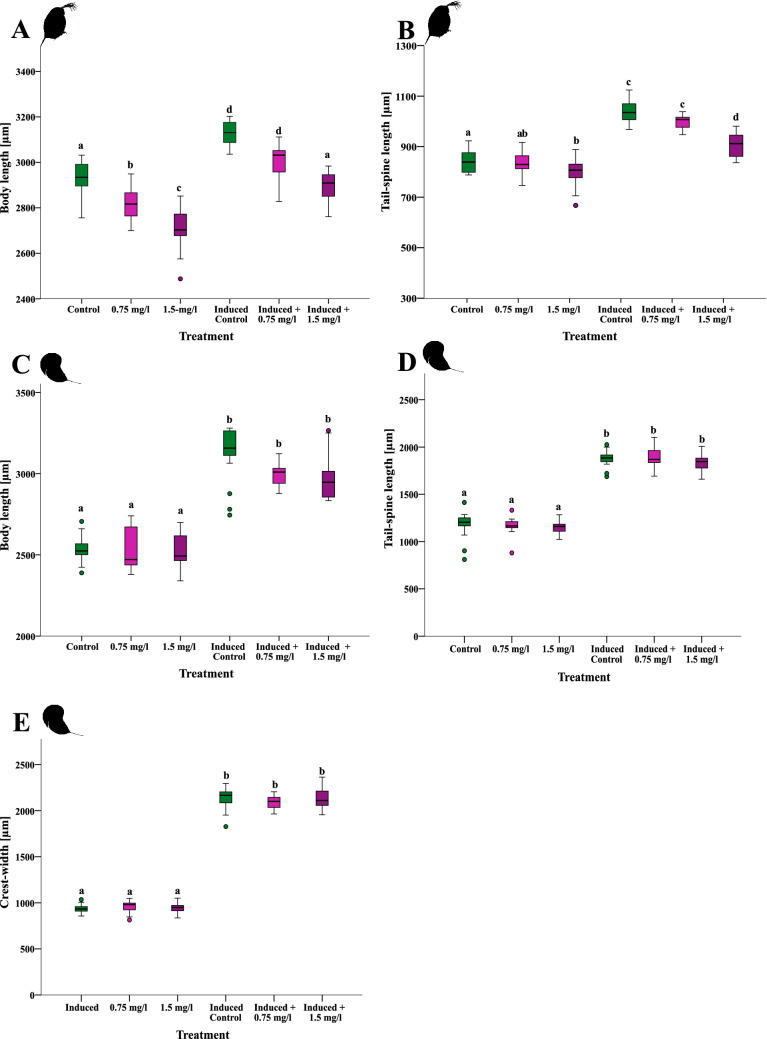
Impact of 2-MNQ on inducible defensive traits *in Daphnia*. (**A**, **B**) Changes in body and tail-spine length in *D. magna* upon *T. cancriformis* and 2-MNQ exposure. (**C**–**E**) Changes in body and tail-spine length and in crest-width in *D. longicephala* upon *Notonecta sp.* and 2-MNQ exposure.

The body length of *D. magna* changed when exposed to either kairomones of *T. cancriformis* or 2-MNQ and to both stressors combined (Fig. 1A; One-way ANOVA with a Tukey-HSD post-hoc Test, *F*_(9,187)_ = 86.735, *P* < 0.001). Further, 2-MNQ shows a concentration depending effect on individuals, because with increasing concentrations of the substance the body length of *D. magna* decreased (*P*_(C-0.75)_ < 0.001; *P*_(C-1.5)_ < 0.001; *P*_(0.75–1.5)_). The presence of kairomones led to an increase in the body length of the animals compared to the control treatment and animals only exposed to 2-MNQ (*P*_(C-I+C)_ < 0.001; *P*_(0.75-I+C)_ < 0.001; *P*_(1.5-I+C)_ < 0.001). When exposed to both stressors together animals still elongated their body length, but with increasing 2-MNQ concentrations the elongation was reduced (*P*_(C-I+0.75)_ < 0.001; *P*_(0.75-I+0.75)_ < 0.001; *P*_(1.5-I+0.75)_ < 0.001; *P*_(0.75-I+1.5)_ = 0.010; *P*_(1.5-I+1.5)_ < 0.001; *P*_(I+C-I+0.75)_ < 0.001; *P*_(I+C-I+1.5)_ < 0.001). When exposed to kairomones and the highest 2-MNQ concentration, the animals were as small as the control animals (*P*_(C-I+1.5)_ = 0.910).

Tail-spine length of *D. magna* differed between the treatments when exposed to either kairomones of *T. cancriformis* or to 2-MNQ and to both stressors combined (Fig. 1B; One-way ANOVA with a Tukey-HSD, *F*_(186,9)_ = 110.711, *P* < 0.001). Further, 2-MNQ had a concentration depending effect on individuals, because only when exposed to the highest concentration of the substance the tail-spine length of *D. magna* decreased (*P*_(C-1.5)_ < 0.001; *P*_(0.75–1.5)_ < 0.121). The presence of kairomones led to an increase in the tail-spine length of the animals compared to the control and the only 2-MNQ exposed animals (*P*_(C-I+C)_ < 0.001; *P*_(0.75-I+C)_ < 0.001; *P*_(1.5-I+C)_ < 0.001). When exposed to both stressors together animals still elongated their tail-spine length, but in the highest concentration of 2-MNQ the elongation was reduced compared to all other treatments (*P*_(C-I+0.75)_ < 0.001; *P*_(0.75-I+0.75)_ < 0.001; *P*_(1.5-I+0.75)_ < 0.001; *P*_(0.75-I+1.5)_ = 0.010; *P*_(1.5-I+1.5)_ < 0.001; *P*_(I+C-I+1.5)_ < 0.001).

The body length of *D. longicephala* differed when exposed to kairomones of *Notonecta spec.* (Fig. 1C; Kruskal–Wallis test with a Bonferroni corrected Dunn’s post hoc test, *H* = 148.496, *P* < 0.001). Treating *D. longicephala* with only 2-MNQ had no effects on the body length of the animals (*P*_(C-0.75)_ = 1.000; *P*_(C-1.5)_ = 1.000). The presence of kairomones led to an increase in the body length of the animals compared to the control treatment and the only 2-MNQ exposed daphnids (*P*_(C-I+C)_ < 0.001; *P*_(0.75-I+C)_ < 0.001; *P*_(1.5-I+C)_ = 0.001). When exposed to both stressors together animals still elongated their body length (*P*_(C-I+0.75)_ = 0.001; *P*_(0.75-I+0.75)_ < 0.001; *P*_(1.5-I+0.75)_ < 0.001; *P*_(0.75-I+1.5)_ = 0.001; *P*_(1.5-I+1.5)_ = 0.001). With increasing 2-MNQ concentrations the body length was reduced, however this was not significant (*P*_(I+C-I+0.75)_ = 1.000; *P*_(I+C-I+1.5)_ = 1.000; *P*_(I+0.75-I+1.5)_ = 1.000).

Tail-spine length of *D. longicephala* differed only when exposed to kairomones of *Notonecta sp.* (Fig. 1D; Kruskal–Wallis test with a Bonferroni corrected Dunn’s post hoc test, *H* = 137.342, *P* < 0.001). While 2-MNQ alone had no effects on the tail-spine length (*P*_(C-0.75)_ = 1.000; *P*_(C-1.5)_ = 1.000; P_(0.75-1.5)_ = 1.000), the presence of kairomones led to an increase in the tail-spine length of the animals compared to the control treatment and the animals only exposed to 2-MNQ (*P*_(C-I+C)_ < 0.001; *P*_(0.75-I+C)_ < 0.001; *P*_(1.5-I+C)_ = 0.007). When exposed to both stressors together animals showed no reduction in their tail-spine length (*P*_(C-I+0.75)_ < 0.001; *P*_(0.75-I+0.75)_ < 0.001; *P*_(1.5-I+0.75) =_ 0.001; *P*_(0.75-I+1.5) =_ 0.001; *P*_(1.5-I+1.5)_ < 0.001).

Crest-width of *D. longicephala* differed when exposed to kairomones of *Notonecta sp.* (Fig. 1E; Kruskal–Wallis test with a Bonferroni corrected Dunn’s post hoc test, *H* = 143.947, *P* < 0.001). 2-MNQ alone had no effects on the expression of the crest-width (*P*_(C-0.75)_ = 1.000; *P*_(C-1.5)_ = 1.000; *P*_(0.75-1.5)_ = 1.000). The presence of kairomones led to an expression of the crest of the animals compared to the control treatment and the animals only exposed to 2-MNQ (*P*_(C-I+C)_ < 0.001; *P*_(0.75-I+C)_ < 0.001; *P*_(1.5-I+C)_ = 0.007). When exposed to both stressors together animals still expressed a crest, whose width was not affected by the presence of 2-MNQ (*P*_(C-I+0.75)_ < 0.001; *P*_(0.75-I+0.75)_ < 0.001; *P*_(1.5-I+0.75)_ = 0.001; *P*_(0.75-I+1.5)_ = 0.001; *P*_(1.5-I+1.5)_ < 0.001; *P*_(I+C-I+0.75)_ < 1.000; *P*_(I+C-I+1.5)_ < 1.000; *P*_(I+0.75-I+1.5)_ < 1.000).

## Discussion

Stressors from different sources are affecting interactions in aquatic habitats and can disturb predator–prey-interactions. In aquatic freshwater ecosystems *Daphnia* species play a major role as keystone species acting as link between primary producers and higher trophic levels^47^. Therefore, they are often used as indicator species to assess the risk of anthropogenic compounds on aquatic ecosystems^48^. In addition, they are textbook examples for the phenomenon of phenotypic plasticity in defensive traits^11,25,49^. It is known that the expression of inducible defences in this genus is sensitive to anthropogenic stressors^50^. However, if the expression of inducible defences is influenced by runoff from invasive terrestrial plants containing allelochemicals as a novel natural stressor, is not known.

In our study both *Daphnia* species responded with an increase in the body- and tail-spine length, when treated with kairomone conditioned media (also containing alarm cues of conspecifics) from their respective predator (Fig. 1A–D). In addition, *D. longicephala* developed a crest, when treated with kairomones from *Notonecta* sp. (Fig. 1E)^51^. These results show that the kairomone conditioned media used in our experiments significantly induced defensive traits in the respective *Daphnia* species. The developed defences from both species have been proven effective against the invertebrate predators we used: It has been shown that an increased body- and tail-spine length protect *D. magna* against the gape-limited predator *T. cancriformis*^19^. The crest and a larger body length in *D. longicephala* protect the animals from the attack of *Notonecta sp.*, since it impedes a firm grasp on the prey and penetration by the sucking apparatus^51,52^.

Our results showed that the exposure to the allelochemical from the invasive plant *I. glandulifera*, 2-MNQ, alone did not induce any defences in the tested species, in contrast to other substances. For instance, endozoan led to the expression of a crest in *D. longicephala*^11^*.* In contrast, *D. magna* individuals solely exposed to 2-MNQ showed a decrease in body and tail-spine length with increasing concentration of 2-MNQ (Fig. 1A, B). This result is in accordance with our former findings and indicates direct toxic effects of the compound^42^. It was shown that naphthoquinones like 2-MNQ can increase the amount of reactive oxygen species (ROS) in cells, which can harm DNA and other cell compartments^53^. Further, naphthoquinones can reduce the activity of ecdysteroids, hormones involved in molting and reproduction in *Daphnia*^40,54^. Furthermore, 2-MNQ may affect energy resources of *Daphnia*, through a reallocation of resources, which will result in an expense of other energy demanding processes or by an enhanced energy uptake^55^. Hence, these direct effects may account for the observed reduced body size and tail-spine length in 2-MNQ exposed *D. magna*^56^. Astonishingly, *D. longicephala* seems to be less sensitive to 2-MNQ since the body length of *D. longicephala* was not reduced when the animals were exposed to the substance, even at higher concentrations (Fig. 1C). This may be explained with a species specific response to stressors^57,58^. It is already known that *Daphnia* species can react differently regarding mortality and reproduction rates, when stressed for example by temperature and humic substances^59,60^.

Nevertheless, when the two *Daphnia* species were simultaneously exposed to 2-MNQ (0.75 and 1.5 mg/l) and kairomone conditioned media, we observed a reduced body length in both species, although only significantly so in *D. magna* (Fig. 1A, C). A smaller body size compared to the animals exposed to predator cues only may interfere with the effectivity of their induced defence, since larger prey are better protected against gape-limited invertebrate predators^61^. For instance, Hunt and Swift showed that larval damselflies preferred smaller *Daphnia pulex* individuals as prey^62^. Thus, smaller and less defended animals may be an easier prey due to a reduced handling time for the predators, which should increase the risk of predation^63^. This may in turn have implications on *Daphnia* populations in natural ecosystems. Further, the elongation of the tail-spine, an effective defence structure in *D. magna* against *T. cancriformis*^19^, was reduced when daphnids were simultaneously exposed to 2-MNQ and kairomone conditioned media (Fig. 1B). Similar to the reduction in body length we cannot differentiate whether 2-MNQ directly interfered with the induction of this defensive trait or whether tail-spine length was reduced due to an overall impact on the fitness of the organism^42^. However, in either way this may increase the risk of a successful attack. The defense mechanism of the elongated tail-spine may function like the 'lizard tail' and thus a larger tail-spine may increase the chance of escape by contact with the predator^64^. In addition, it has been shown that inducible morphological defensive traits are adapted to predator and conspecific density on a fine scale and therefore a reduced expression of the defensive trait may be maladaptive and enhance predation risk^22,65^.

In contrast, in *D. longicephala* the elongation of the tail-spine length and the formation of the crest were not affected by the allelochemicals (Fig. 1D and E).

Thus, *D. magna* individuals exposed to 2-MNQ may be an easier prey due to their reduced defences, while *D. longicephala* still could defend itself against its predator. Future studies will have to show whether these effects are mediated directly by interference with induced defence mechanisms or a general negative effect on the fitness of *D. magna*. These differential sensitivities towards allelochemicals may result in shifts in *Daphnia* communities in freshwater ecosystems due to an increased predation on less defended animals, e. g. calcium reduction led to less defended *Daphnia* species compared to a less calcium depending competitor, the cladoceran *Holopedium glacialis*, and thus to a replacement in the ecosystem^21,66^.

In summary, we show that the allelochemical 2-MNQ released by *I. glandulifera* reduces the defensibility in *Daphnia,* but depending on the sensitivity of species towards the chemical stressor. Studies with other chemical stressors, such as copper showed that daphnids can develop a higher resistance towards copper over time due to strong selection pressure^67^. Hence it is possible that *Daphnia* populations may adapt to certain concentrations of 2-MNQ and thus the risk posed by allelochemicals released from *I. glandulifera* may decline. However, phytotoxins, such as alkaloids or quinones affect daphnids already at relatively low concentrations^30,37,41,42^. In addition, naphthoquinones have high persistence time in pond water, e. g. juglone was shown to have a half-life of 87 ± 7.4 h^68^. Thus, allelochemicals released from *I. glandulifera* may affect the fitness of *Daphnia* due to a longer persistence in the aquatic ecosystem. In this context, our results suggest that leachate containing 2-MNQ from *I. glandulifera* in water bodies increases the susceptibility of *Daphnia* towards invertebrate predators due to reduced defensibility. As daphnids are keystone species in lentic ecosystem, the decline of their populations, by a higher predation efficacy, may affect food web dynamics. Since *I. glandulifera* is often forming monospecific stands at riparian zones our results highlight the importance to also evaluate the risks of terrestrial invasive plants on aquatic ecosystems.

## Supplementary Information

Supplementary Information.

## Data Availability

Raw data for Posthoc tests and Fig. 1 is placed in supplemental data, Tables S1–S4.
