# Supplementary material for: Allelochemical run-off from the invasive terrestrial plant Impatiens glandulifera decreases defensibility in Daphnia
Source: Sci Rep. 2023 Jan 21;13:1207. doi: 10.1038/s41598-023-27667-4 (PMC9867768; doi:10.1038/s41598-023-27667-4)
Supplement: Supplementary file 1 — Supplementary Information. [file 41598_2023_27667_MOESM1_ESM.docx]

**Supplementary Information:** Allelochemical run-off from the invasive terrestrial plant *Impatiens glandulifera* decreases defensibility in *Daphnia*.

Authors: Jens G. P. Diller^1.2^. Frederic Hüftlein^1^. Darleen Lücker^1^. Heike Feldhaar^1.2.3.*^ and Christian Laforsch^1.2. 3.*^

^1^Animal Ecology I. Universitaetsstraße 30. 95447 Bayreuth

^2^Bayreuth Center for Ecology and Environmental Research (BayCEER). Universitaetsstraße 30. 95447 Bayreuth


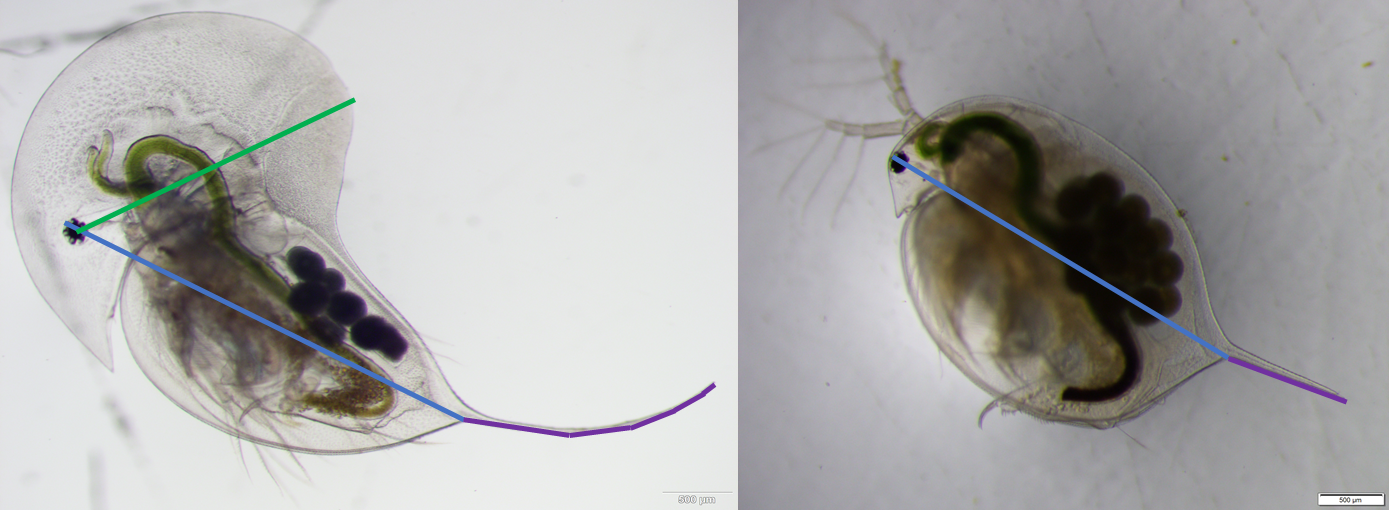


**Figure S1: Morphological measurements of Daphnia longicephala (left) and Daphnia magna (right):** The body length was measured from the base of the tail-spine to the anterior end of the compound eye (blue line). The crestwidth for D. longicephala was measured from the left margin of the compound eye to the right lateral margin of the head (green line). The tail-spine length for D. magna was measured from the base to its tip (purple line). Unlike the straight tail spine of D. magna the tail spine of D. longicephala grows in a curve. and therefore a polygonal line along the tail-spine was used. The crest height was calculated by subtracting the body length from the total length (purple line).

**Table S1: Post hoc tests for measured bodylength of D. longicephala at age of maturity.** C (control); 0.075 mg/l 2-MNQ concentration; 0.75 mg/l 2-MNQ concentration; 1.5 mg/l 2-MNQ concentration and the corresponding DMSO controls and the Notonecta kairomone treatments. Asymptotic significances (2-sided tests) are displayed. The significance level is .05.

| **Pairwise Comparisons of Treatment** | | | | | |
| --- | --- | --- | --- | --- | --- |
| Sample 1-Sample 2 | Test Statistic | Std. Error | Std. Test Statistic | Sig. | Adj. Sig. |
| 2-MNQ-0.75-Control-2-MNQ-1.5-Control | -1.463 | 17.803 | -.082 | .934 | 1.000 |
| 2-MNQ-0.75-Control-DMSO-0.75-Control | 5.306 | 18.042 | .294 | .769 | 1.000 |
| 2-MNQ-0.75-Control-Control | 6.832 | 17.803 | .384 | .701 | 1.000 |
| 2-MNQ-0.75-Control-DMSO-1.5-Control | 15.463 | 17.803 | .869 | .385 | 1.000 |
| 2-MNQ-0.75-Control-2-MNQ-1.5-Notonecta | -79.129 | 18.305 | -4.323 | .000 | .001 |
| 2-MNQ-0.75-Control-2-MNQ-0.75-Notonecta | -79.356 | 17.585 | -4.513 | .000 | .000 |
| 2-MNQ-0.75-Control-Notonecta | -109.194 | 18.042 | -6.052 | .000 | .000 |
| 2-MNQ-0.75-Control-DMSO-0.75-Notonecta | -110.906 | 17.585 | -6.307 | .000 | .000 |
| 2-MNQ-0.75-Control-DMSO-1.5-Notonecta | -116.411 | 17.803 | -6.539 | .000 | .000 |
| 2-MNQ-1.5-Control-DMSO-0.75-Control | 3.842 | 17.803 | .216 | .829 | 1.000 |
| 2-MNQ-1.5-Control-Control | 5.368 | 17.561 | .306 | .760 | 1.000 |
| 2-MNQ-1.5-Control-DMSO-1.5-Control | 14.000 | 17.561 | .797 | .425 | 1.000 |
| 2-MNQ-1.5-Control-2-MNQ-1.5-Notonecta | -77.666 | 18.070 | -4.298 | .000 | .001 |
| 2-MNQ-1.5-Control-2-MNQ-0.75-Notonecta | -77.892 | 17.340 | -4.492 | .000 | .000 |
| 2-MNQ-1.5-Control-Notonecta | -107.731 | 17.803 | -6.051 | .000 | .000 |
| 2-MNQ-1.5-Control-DMSO-0.75-Notonecta | -109.442 | 17.340 | -6.312 | .000 | .000 |
| 2-MNQ-1.5-Control-DMSO-1.5-Notonecta | -114.947 | 17.561 | -6.546 | .000 | .000 |
| DMSO-0.75-Control-Control | 1.526 | 17.803 | .086 | .932 | 1.000 |
| DMSO-0.75-Control-DMSO-1.5-Control | -10.158 | 17.803 | -.571 | .568 | 1.000 |
| DMSO-0.75-Control-2-MNQ-1.5-Notonecta | -73.824 | 18.305 | -4.033 | .000 | .002 |
| DMSO-0.75-Control-2-MNQ-0.75-Notonecta | -74.050 | 17.585 | -4.211 | .000 | .001 |
| DMSO-0.75-Control-Notonecta | -103.889 | 18.042 | -5.758 | .000 | .000 |
| DMSO-0.75-Control-DMSO-0.75-Notonecta | -105.600 | 17.585 | -6.005 | .000 | .000 |
| DMSO-0.75-Control-DMSO-1.5-Notonecta | -111.105 | 17.803 | -6.241 | .000 | .000 |
| Control-DMSO-1.5-Control | -8.632 | 17.561 | -.492 | .623 | 1.000 |
| Control-2-MNQ-1.5-Notonecta | -72.297 | 18.070 | -4.001 | .000 | .003 |
| Control-2-MNQ-0.75-Notonecta | -72.524 | 17.340 | -4.182 | .000 | .001 |
| Control-Notonecta | -102.363 | 17.803 | -5.750 | .000 | .000 |
| Control-DMSO-0.75-Notonecta | -104.074 | 17.340 | -6.002 | .000 | .000 |
| Control-DMSO-1.5-Notonecta | -109.579 | 17.561 | -6.240 | .000 | .000 |
| DMSO-1.5-Control-2-MNQ-1.5-Notonecta | -63.666 | 18.070 | -3.523 | .000 | .019 |
| DMSO-1.5-Control-2-MNQ-0.75-Notonecta | -63.892 | 17.340 | -3.685 | .000 | .010 |
| DMSO-1.5-Control-Notonecta | -93.731 | 17.803 | -5.265 | .000 | .000 |
| DMSO-1.5-Control-DMSO-0.75-Notonecta | -95.442 | 17.340 | -5.504 | .000 | .000 |
| DMSO-1.5-Control-DMSO-1.5-Notonecta | -100.947 | 17.561 | -5.748 | .000 | .000 |
| 2-MNQ-1.5-Notonecta-2-MNQ-0.75-Notonecta | .226 | 17.855 | .013 | .990 | 1.000 |
| 2-MNQ-1.5-Notonecta-Notonecta | 30.065 | 18.305 | 1.642 | .100 | 1.000 |
| 2-MNQ-1.5-Notonecta-DMSO-0.75-Notonecta | 31.776 | 17.855 | 1.780 | .075 | 1.000 |
| 2-MNQ-1.5-Notonecta-DMSO-1.5-Notonecta | 37.282 | 18.070 | 2.063 | .039 | 1.000 |
| 2-MNQ-0.75-Notonecta-Notonecta | 29.839 | 17.585 | 1.697 | .090 | 1.000 |
| 2-MNQ-0.75-Notonecta-DMSO-0.75-Notonecta | 31.550 | 17.116 | 1.843 | .065 | 1.000 |
| 2-MNQ-0.75-Notonecta-DMSO-1.5-Notonecta | 37.055 | 17.340 | 2.137 | .033 | 1.000 |
| Notonecta-DMSO-0.75-Notonecta | -1.711 | 17.585 | -.097 | .922 | 1.000 |
| Notonecta-DMSO-1.5-Notonecta | -7.216 | 17.803 | -.405 | .685 | 1.000 |
| DMSO-0.75-Notonecta-DMSO-1.5-Notonecta | -5.505 | 17.340 | -.317 | .751 | 1.000 |
|  | | | | | |

**Table S2: Post Hoc tests for total spinelength of D. longicephala at age at maturity:** C (control); 0.075 mg/l 2-MNQ concentration; 0.75 mg/l 2-MNQ concentration; 1.5 mg/l 2-MNQ concentration and the corresponding DMSO controls and the Notonecta kairomone treatments. Asymptotic significances (2-sided tests) are displayed. The significance level is .05.

| **Pairwise Comparisons of Treatment** | | | | | |
| --- | --- | --- | --- | --- | --- |
| Sample 1-Sample 2 | Test Statistic | Std. Error | Std. Test Statistic | Sig. | Adj. Sig. |
| DMSO-0.75-Control-2-MNQ-1.5-Control | -10.778 | 17.080 | -.631 | .528 | 1.000 |
| DMSO-0.75-Control-2-MNQ-0.75-Control | -17.626 | 17.329 | -1.017 | .309 | 1.000 |
| DMSO-0.75-Control-DMSO-1.5-Control | -18.889 | 17.080 | -1.106 | .269 | 1.000 |
| DMSO-0.75-Control-Control | 25.302 | 17.329 | 1.460 | .144 | 1.000 |
| DMSO-0.75-Control-DMSO-0.75-Notonecta | -90.572 | 16.853 | -5.374 | .000 | .000 |
| DMSO-0.75-Control-2-MNQ-1.5-Notonecta | -97.714 | 17.329 | -5.639 | .000 | .000 |
| DMSO-0.75-Control-DMSO-1.5-Notonecta | -106.174 | 17.605 | -6.031 | .000 | .000 |
| DMSO-0.75-Control-Notonecta | -109.920 | 17.329 | -6.343 | .000 | .000 |
| DMSO-0.75-Control-2-MNQ-0.75-Notonecta | -110.261 | 16.647 | -6.623 | .000 | .000 |
| 2-MNQ-1.5-Control-2-MNQ-0.75-Control | 6.848 | 17.329 | .395 | .693 | 1.000 |
| 2-MNQ-1.5-Control-DMSO-1.5-Control | 8.111 | 17.080 | .475 | .635 | 1.000 |
| 2-MNQ-1.5-Control-Control | 14.525 | 17.329 | .838 | .402 | 1.000 |
| 2-MNQ-1.5-Control-DMSO-0.75-Notonecta | -79.794 | 16.853 | -4.735 | .000 | .000 |
| 2-MNQ-1.5-Control-2-MNQ-1.5-Notonecta | -86.936 | 17.329 | -5.017 | .000 | .000 |
| 2-MNQ-1.5-Control-DMSO-1.5-Notonecta | -95.396 | 17.605 | -5.419 | .000 | .000 |
| 2-MNQ-1.5-Control-Notonecta | -99.142 | 17.329 | -5.721 | .000 | .000 |
| 2-MNQ-1.5-Control-2-MNQ-0.75-Notonecta | -99.483 | 16.647 | -5.976 | .000 | .000 |
| 2-MNQ-0.75-Control-DMSO-1.5-Control | 1.263 | 17.329 | .073 | .942 | 1.000 |
| 2-MNQ-0.75-Control-Control | 7.676 | 17.575 | .437 | .662 | 1.000 |
| 2-MNQ-0.75-Control-DMSO-0.75-Notonecta | -72.946 | 17.106 | -4.264 | .000 | .001 |
| 2-MNQ-0.75-Control-2-MNQ-1.5-Notonecta | -80.088 | 17.575 | -4.557 | .000 | .000 |
| 2-MNQ-0.75-Control-DMSO-1.5-Notonecta | -88.548 | 17.847 | -4.961 | .000 | .000 |
| 2-MNQ-0.75-Control-Notonecta | -92.294 | 17.575 | -5.251 | .000 | .000 |
| 2-MNQ-0.75-Control-2-MNQ-0.75-Notonecta | -92.635 | 16.903 | -5.480 | .000 | .000 |
| DMSO-1.5-Control-Control | 6.413 | 17.329 | .370 | .711 | 1.000 |
| DMSO-1.5-Control-DMSO-0.75-Notonecta | -71.683 | 16.853 | -4.253 | .000 | .001 |
| DMSO-1.5-Control-2-MNQ-1.5-Notonecta | -78.825 | 17.329 | -4.549 | .000 | .000 |
| DMSO-1.5-Control-DMSO-1.5-Notonecta | -87.285 | 17.605 | -4.958 | .000 | .000 |
| DMSO-1.5-Control-Notonecta | -91.031 | 17.329 | -5.253 | .000 | .000 |
| DMSO-1.5-Control-2-MNQ-0.75-Notonecta | -91.372 | 16.647 | -5.489 | .000 | .000 |
| Control-DMSO-0.75-Notonecta | -65.269 | 17.106 | -3.816 | .000 | .006 |
| Control-2-MNQ-1.5-Notonecta | -72.412 | 17.575 | -4.120 | .000 | .002 |
| Control-DMSO-1.5-Notonecta | -80.871 | 17.847 | -4.531 | .000 | .000 |
| Control-Notonecta | -84.618 | 17.575 | -4.815 | .000 | .000 |
| Control-2-MNQ-0.75-Notonecta | -84.959 | 16.903 | -5.026 | .000 | .000 |
| DMSO-0.75-Notonecta-2-MNQ-1.5-Notonecta | -7.142 | 17.106 | -.418 | .676 | 1.000 |
| DMSO-0.75-Notonecta-DMSO-1.5-Notonecta | -15.602 | 17.386 | -.897 | .370 | 1.000 |
| DMSO-0.75-Notonecta-Notonecta | 19.348 | 17.106 | 1.131 | .258 | 1.000 |
| DMSO-0.75-Notonecta-2-MNQ-0.75-Notonecta | -19.689 | 16.415 | -1.199 | .230 | 1.000 |
| 2-MNQ-1.5-Notonecta-DMSO-1.5-Notonecta | 8.460 | 17.847 | .474 | .636 | 1.000 |
| 2-MNQ-1.5-Notonecta-Notonecta | 12.206 | 17.575 | .695 | .487 | 1.000 |
| 2-MNQ-1.5-Notonecta-2-MNQ-0.75-Notonecta | 12.547 | 16.903 | .742 | .458 | 1.000 |
| DMSO-1.5-Notonecta-Notonecta | 3.746 | 17.847 | .210 | .834 | 1.000 |
| DMSO-1.5-Notonecta-2-MNQ-0.75-Notonecta | -4.088 | 17.186 | -.238 | .812 | 1.000 |
| Notonecta-2-MNQ-0.75-Notonecta | -.341 | 16.903 | -.020 | .984 | 1.000 |
|  | | | | | |

**Table S3: Post hoc tests for measured bodylength of D. magna at age of maturity.** C (control); 0.075 mg/l 2-MNQ concentration; 0.75 mg/l 2-MNQ concentration; 1.5 mg/l 2-MNQ concentration and the corresponding DMSO controls and the Triops cancriformis kairomone treatments. Asymptotic significances (2-sided tests) are displayed. The significance level is .05.

|  | | | | | | | |
| --- | --- | --- | --- | --- | --- | --- | --- |
| Variable | | | Mean difference (I-J) | Mean error | Significance | 95%-Confidence interval | |
|  |  |  |  |  |  | Lower level | Upper level |
| Bodylength[µm] | 0.75-2-MNQ | 0.75-DMSO | -122.67915^*^ | 21.37113 | .000 | -191.1166 | -54.2417 |
|  |  | 1.5-2-MNQ | 107.90569^*^ | 21.65050 | .000 | 38.5736 | 177.2377 |
|  |  | 1.5-DMSO | -114.10160^*^ | 21.37113 | .000 | -182.5390 | -45.6642 |
|  |  | Control | -110.61925^*^ | 21.65050 | .000 | -179.9513 | -41.2872 |
|  |  | Triops-0.75-2-MNQ | -190.11952^*^ | 21.65050 | .000 | -259.4516 | -120.7875 |
|  |  | Triops-0.75-DMSO | -326.01905^*^ | 21.37113 | .000 | -394.4565 | -257.5816 |
|  |  | Triops-1.5-DMSO | -312.96940^*^ | 21.37113 | .000 | -381.4068 | -244.5320 |
|  |  | Triops-1.5-2-MNQ | -79.25215^*^ | 21.37113 | .010 | -147.6896 | -10.8147 |
|  |  | Triops-Control | -308.38485^*^ | 21.37113 | .000 | -376.8223 | -239.9474 |
|  | 0.75-DMSO | 0.75-2-MNQ | 122.67915^*^ | 21.37113 | .000 | 54.2417 | 191.1166 |
|  |  | 1.5-2-MNQ | 230.58484^*^ | 21.65050 | .000 | 161.2528 | 299.9169 |
|  |  | 1.5-DMSO | 8.57755 | 21.37113 | 1.000 | -59.8599 | 77.0150 |
|  |  | Control | 12.05990 | 21.65050 | 1.000 | -57.2722 | 81.3919 |
|  |  | Triops-0.75-2-MNQ | -67.44037 | 21.65050 | .064 | -136.7724 | 1.8917 |
|  |  | Triops-0.75-DMSO | -203.33990^*^ | 21.37113 | .000 | -271.7773 | -134.9025 |
|  |  | Triops-1.5-DMSO | -190.29025^*^ | 21.37113 | .000 | -258.7277 | -121.8528 |
|  |  | Triops-1.5-2-MNQ | 43.42700 | 21.37113 | .578 | -25.0104 | 111.8644 |
|  |  | Triops-Control | -185.70570^*^ | 21.37113 | .000 | -254.1431 | -117.2683 |
|  | 1.5-2-MNQ | 0.75-2-MNQ | -107.90569^*^ | 21.65050 | .000 | -177.2377 | -38.5736 |
|  |  | 0.75-DMSO | -230.58484^*^ | 21.65050 | .000 | -299.9169 | -161.2528 |
|  |  | 1.5-DMSO | -222.00729^*^ | 21.65050 | .000 | -291.3393 | -152.6752 |
|  |  | Control | -218.52495^*^ | 21.92631 | .000 | -288.7402 | -148.3097 |
|  |  | Triops-0.75-2-MNQ | -298.02521^*^ | 21.92631 | .000 | -368.2405 | -227.8099 |
|  |  | Triops-0.75-DMSO | -433.92474^*^ | 21.65050 | .000 | -503.2568 | -364.5927 |
|  |  | Triops-1.5-DMSO | -420.87509^*^ | 21.65050 | .000 | -490.2071 | -351.5430 |
|  |  | Triops-1.5-2-MNQ | -187.15784^*^ | 21.65050 | .000 | -256.4899 | -117.8258 |
|  |  | Triops-Control | -416.29054^*^ | 21.65050 | .000 | -485.6226 | -346.9585 |
|  | 1.5-DMSO | 0.75-2-MNQ | 114.10160^*^ | 21.37113 | .000 | 45.6642 | 182.5390 |
|  |  | 0.75-DMSO | -8.57755 | 21.37113 | 1.000 | -77.0150 | 59.8599 |
|  |  | 1.5-2-MNQ | 222.00729^*^ | 21.65050 | .000 | 152.6752 | 291.3393 |
|  |  | Control | 3.48235 | 21.65050 | 1.000 | -65.8497 | 72.8144 |
|  |  | Triops-0.75-2-MNQ | -76.01792^*^ | 21.65050 | .019 | -145.3500 | -6.6859 |
|  |  | Triops-0.75-DMSO | -211.91745^*^ | 21.37113 | .000 | -280.3549 | -143.4800 |
|  |  | Triops-1.5-DMSO | -198.86780^*^ | 21.37113 | .000 | -267.3052 | -130.4304 |
|  |  | Triops-1.5-2-MNQ | 34.84945 | 21.37113 | .831 | -33.5880 | 103.2869 |
|  |  | Triops-Control | -194.28325^*^ | 21.37113 | .000 | -262.7207 | -125.8458 |
|  | Control | 0.75-2-MNQ | 110.61925^*^ | 21.65050 | .000 | 41.2872 | 179.9513 |
|  |  | 0.75-DMSO | -12.05990 | 21.65050 | 1.000 | -81.3919 | 57.2722 |
|  |  | 1.5-2-MNQ | 218.52495^*^ | 21.92631 | .000 | 148.3097 | 288.7402 |
|  |  | 1.5-DMSO | -3.48235 | 21.65050 | 1.000 | -72.8144 | 65.8497 |
|  |  | Triops-0.75-2-MNQ | -79.50026^*^ | 21.92631 | .013 | -149.7156 | -9.2850 |
|  |  | Triops-0.75-DMSO | -215.39980^*^ | 21.65050 | .000 | -284.7318 | -146.0677 |
|  |  | Triops-1.5-DMSO | -202.35015^*^ | 21.65050 | .000 | -271.6822 | -133.0181 |
|  |  | Triops-1.5-2-MNQ | 31.36710 | 21.65050 | .910 | -37.9649 | 100.6992 |
|  |  | Triops-Control | -197.76560^*^ | 21.65050 | .000 | -267.0976 | -128.4335 |
|  | Triops-0.75-2-MNQ | 0.75-2-MNQ | 190.11952^*^ | 21.65050 | .000 | 120.7875 | 259.4516 |
|  |  | 0.75-DMSO | 67.44037 | 21.65050 | .064 | -1.8917 | 136.7724 |
|  |  | 1.5-2-MNQ | 298.02521^*^ | 21.92631 | .000 | 227.8099 | 368.2405 |
|  |  | 1.5-DMSO | 76.01792^*^ | 21.65050 | .019 | 6.6859 | 145.3500 |
|  |  | Control | 79.50026^*^ | 21.92631 | .013 | 9.2850 | 149.7156 |
|  |  | Triops-0.75-DMSO | -135.89953^*^ | 21.65050 | .000 | -205.2316 | -66.5675 |
|  |  | Triops-1.5-DMSO | -122.84988^*^ | 21.65050 | .000 | -192.1819 | -53.5178 |
|  |  | Triops-1.5-2-MNQ | 110.86737^*^ | 21.65050 | .000 | 41.5353 | 180.1994 |
|  |  | Triops-Control | -118.26533^*^ | 21.65050 | .000 | -187.5974 | -48.9333 |
|  | Triops-0.75-DMSO | 0.75-2-MNQ | 326.01905^*^ | 21.37113 | .000 | 257.5816 | 394.4565 |
|  |  | 0.75-DMSO | 203.33990^*^ | 21.37113 | .000 | 134.9025 | 271.7773 |
|  |  | 1.5-2-MNQ | 433.92474^*^ | 21.65050 | .000 | 364.5927 | 503.2568 |
|  |  | 1.5-DMSO | 211.91745^*^ | 21.37113 | .000 | 143.4800 | 280.3549 |
|  |  | Control | 215.39980^*^ | 21.65050 | .000 | 146.0677 | 284.7318 |
|  |  | Triops-0.75-2-MNQ | 135.89953^*^ | 21.65050 | .000 | 66.5675 | 205.2316 |
|  |  | Triops-1.5-DMSO | 13.04965 | 21.37113 | 1.000 | -55.3878 | 81.4871 |
|  |  | Triops-1.5-2-MNQ | 246.76690^*^ | 21.37113 | .000 | 178.3295 | 315.2043 |
|  |  | Triops-Control | 17.63420 | 21.37113 | .998 | -50.8032 | 86.0716 |
|  | Triops-1.5-DMSO | 0.75-2-MNQ | 312.96940^*^ | 21.37113 | .000 | 244.5320 | 381.4068 |
|  |  | 0.75-DMSO | 190.29025^*^ | 21.37113 | .000 | 121.8528 | 258.7277 |
|  |  | 1.5-2-MNQ | 420.87509^*^ | 21.65050 | .000 | 351.5430 | 490.2071 |
|  |  | 1.5-DMSO | 198.86780^*^ | 21.37113 | .000 | 130.4304 | 267.3052 |
|  |  | Control | 202.35015^*^ | 21.65050 | .000 | 133.0181 | 271.6822 |
|  |  | Triops-0.75-2-MNQ | 122.84988^*^ | 21.65050 | .000 | 53.5178 | 192.1819 |
|  |  | Triops-0.75-DMSO | -13.04965 | 21.37113 | 1.000 | -81.4871 | 55.3878 |
|  |  | Triops-1.5-2-MNQ | 233.71725^*^ | 21.37113 | .000 | 165.2798 | 302.1547 |
|  |  | Triops-Control | 4.58455 | 21.37113 | 1.000 | -63.8529 | 73.0220 |
|  | Triops-1.5-2-MNQ | 0.75-2-MNQ | 79.25215^*^ | 21.37113 | .010 | 10.8147 | 147.6896 |
|  |  | 0.75-DMSO | -43.42700 | 21.37113 | .578 | -111.8644 | 25.0104 |
|  |  | 1.5-2-MNQ | 187.15784^*^ | 21.65050 | .000 | 117.8258 | 256.4899 |
|  |  | 1.5-DMSO | -34.84945 | 21.37113 | .831 | -103.2869 | 33.5880 |
|  |  | Control | -31.36710 | 21.65050 | .910 | -100.6992 | 37.9649 |
|  |  | Triops-0.75-2-MNQ | -110.86737^*^ | 21.65050 | .000 | -180.1994 | -41.5353 |
|  |  | Triops-0.75-DMSO | -246.76690^*^ | 21.37113 | .000 | -315.2043 | -178.3295 |
|  |  | Triops-1.5-DMSO | -233.71725^*^ | 21.37113 | .000 | -302.1547 | -165.2798 |
|  |  | Triops-Control | -229.13270^*^ | 21.37113 | .000 | -297.5701 | -160.6953 |
|  | Triops-Control | 0.75-2-MNQ | 308.38485^*^ | 21.37113 | .000 | 239.9474 | 376.8223 |
|  |  | 0.75-DMSO | 185.70570^*^ | 21.37113 | .000 | 117.2683 | 254.1431 |
|  |  | 1.5-2-MNQ | 416.29054^*^ | 21.65050 | .000 | 346.9585 | 485.6226 |
|  |  | 1.5-DMSO | 194.28325^*^ | 21.37113 | .000 | 125.8458 | 262.7207 |
|  |  | Control | 197.76560^*^ | 21.65050 | .000 | 128.4335 | 267.0976 |
|  |  | Triops-0.75-2-MNQ | 118.26533^*^ | 21.65050 | .000 | 48.9333 | 187.5974 |
|  |  | Triops-0.75-DMSO | -17.63420 | 21.37113 | .998 | -86.0716 | 50.8032 |
|  |  | Triops-1.5-DMSO | -4.58455 | 21.37113 | 1.000 | -73.0220 | 63.8529 |
|  |  | Triops-1.5-2-MNQ | 229.13270^*^ | 21.37113 | .000 | 160.6953 | 297.5701 |

**Table S4: Post hoc tests for measured spinelength of D. magna at age of maturity.** C (control); 0.075 mg/l 2-MNQ concentration; 0.75 mg/l 2-MNQ concentration; 1.5 mg/l 2-MNQ concentration and the corresponding DMSO controls and the Triops cancriformis kairomone treatments. Asymptotic significances (2-sided tests) are displayed. The significance level is .05.

| Variable | | | Mean difference (I-J) | Mean error | Significance | 95%-Confidence interval | |
| --- | --- | --- | --- | --- | --- | --- | --- |
|  |  |  |  |  |  | Lower level | Upper level |
| Spinelength[µm] | 0.75-2-MNQ | 0.75-DMSO | -5.03935 | 13.74547 | 1.000 | -49.0598 | 38.9811 |
|  |  | 1.5-2-MNQ | 40.01458 | 13.92516 | .121 | -4.5813 | 84.6104 |
|  |  | 1.5-DMSO | 2.19327 | 13.92516 | 1.000 | -42.4026 | 46.7891 |
|  |  | Control | -6.34663 | 13.92516 | 1.000 | -50.9425 | 38.2492 |
|  |  | Triops-0.75-2-MNQ | -163.79457^*^ | 13.92516 | .000 | -208.3904 | -119.1987 |
|  |  | Triops-0.75-DMSO | -220.31860^*^ | 13.74547 | .000 | -264.3390 | -176.2982 |
|  |  | Triops-1.5-DMSO | -204.63995^*^ | 13.74547 | .000 | -248.6604 | -160.6195 |
|  |  | Triops-1.5-2-MNQ | -73.18755^*^ | 13.74547 | .000 | -117.2080 | -29.1671 |
|  |  | Triops-Control | -202.91245^*^ | 13.74547 | .000 | -246.9329 | -158.8920 |
|  | 0.75-DMSO | 0.75-2-MNQ | 5.03935 | 13.74547 | 1.000 | -38.9811 | 49.0598 |
|  |  | 1.5-2-MNQ | 45.05393^*^ | 13.92516 | .045 | .4581 | 89.6498 |
|  |  | 1.5-DMSO | 7.23262 | 13.92516 | 1.000 | -37.3632 | 51.8285 |
|  |  | Control | -1.30728 | 13.92516 | 1.000 | -45.9031 | 43.2886 |
|  |  | Triops-0.75-2-MNQ | -158.75522^*^ | 13.92516 | .000 | -203.3511 | -114.1594 |
|  |  | Triops-0.75-DMSO | -215.27925^*^ | 13.74547 | .000 | -259.2997 | -171.2588 |
|  |  | Triops-1.5-DMSO | -199.60060^*^ | 13.74547 | .000 | -243.6210 | -155.5802 |
|  |  | Triops-1.5-2-MNQ | -68.14820^*^ | 13.74547 | .000 | -112.1686 | -24.1278 |
|  |  | Triops-Control | -197.87310^*^ | 13.74547 | .000 | -241.8935 | -153.8527 |
|  | 1.5-2-MNQ | 0.75-2-MNQ | -40.01458 | 13.92516 | .121 | -84.6104 | 4.5813 |
|  |  | 0.75-DMSO | -45.05393^*^ | 13.92516 | .045 | -89.6498 | -.4581 |
|  |  | 1.5-DMSO | -37.82132 | 14.10256 | .189 | -82.9853 | 7.3427 |
|  |  | Control | -46.36121^*^ | 14.10256 | .039 | -91.5252 | -1.1972 |
|  |  | Triops-0.75-2-MNQ | -203.80916^*^ | 14.10256 | .000 | -248.9731 | -158.6452 |
|  |  | Triops-0.75-DMSO | -260.33318^*^ | 13.92516 | .000 | -304.9290 | -215.7373 |
|  |  | Triops-1.5-DMSO | -244.65453^*^ | 13.92516 | .000 | -289.2504 | -200.0587 |
|  |  | Triops-1.5-2-MNQ | -113.20213^*^ | 13.92516 | .000 | -157.7980 | -68.6063 |
|  |  | Triops-Control | -242.92703^*^ | 13.92516 | .000 | -287.5229 | -198.3312 |
|  | 1.5-DMSO | 0.75-2-MNQ | -2.19327 | 13.92516 | 1.000 | -46.7891 | 42.4026 |
|  |  | 0.75-DMSO | -7.23262 | 13.92516 | 1.000 | -51.8285 | 37.3632 |
|  |  | 1.5-2-MNQ | 37.82132 | 14.10256 | .189 | -7.3427 | 82.9853 |
|  |  | Control | -8.53989 | 14.10256 | 1.000 | -53.7039 | 36.6241 |
|  |  | Triops-0.75-2-MNQ | -165.98784^*^ | 14.10256 | .000 | -211.1518 | -120.8239 |
|  |  | Triops-0.75-DMSO | -222.51187^*^ | 13.92516 | .000 | -267.1077 | -177.9160 |
|  |  | Triops-1.5-DMSO | -206.83322^*^ | 13.92516 | .000 | -251.4291 | -162.2374 |
|  |  | Triops-1.5-2-MNQ | -75.38082^*^ | 13.92516 | .000 | -119.9767 | -30.7850 |
|  |  | Triops-Control | -205.10572^*^ | 13.92516 | .000 | -249.7016 | -160.5099 |
|  | Control | 0.75-2-MNQ | 6.34663 | 13.92516 | 1.000 | -38.2492 | 50.9425 |
|  |  | 0.75-DMSO | 1.30728 | 13.92516 | 1.000 | -43.2886 | 45.9031 |
|  |  | 1.5-2-MNQ | 46.36121^*^ | 14.10256 | .039 | 1.1972 | 91.5252 |
|  |  | 1.5-DMSO | 8.53989 | 14.10256 | 1.000 | -36.6241 | 53.7039 |
|  |  | Triops-0.75-2-MNQ | -157.44795^*^ | 14.10256 | .000 | -202.6119 | -112.2840 |
|  |  | Triops-0.75-DMSO | -213.97197^*^ | 13.92516 | .000 | -258.5678 | -169.3761 |
|  |  | Triops-1.5-DMSO | -198.29332^*^ | 13.92516 | .000 | -242.8892 | -153.6975 |
|  |  | Triops-1.5-2-MNQ | -66.84092^*^ | 13.92516 | .000 | -111.4368 | -22.2451 |
|  |  | Triops-Control | -196.56582^*^ | 13.92516 | .000 | -241.1617 | -151.9700 |
|  | Triops-0.75-2-MNQ | 0.75-2-MNQ | 163.79457^*^ | 13.92516 | .000 | 119.1987 | 208.3904 |
|  |  | 0.75-DMSO | 158.75522^*^ | 13.92516 | .000 | 114.1594 | 203.3511 |
|  |  | 1.5-2-MNQ | 203.80916^*^ | 14.10256 | .000 | 158.6452 | 248.9731 |
|  |  | 1.5-DMSO | 165.98784^*^ | 14.10256 | .000 | 120.8239 | 211.1518 |
|  |  | Control | 157.44795^*^ | 14.10256 | .000 | 112.2840 | 202.6119 |
|  |  | Triops-0.75-DMSO | -56.52403^*^ | 13.92516 | .003 | -101.1199 | -11.9282 |
|  |  | Triops-1.5-DMSO | -40.84538 | 13.92516 | .104 | -85.4412 | 3.7505 |
|  |  | Triops-1.5-2-MNQ | 90.60702^*^ | 13.92516 | .000 | 46.0112 | 135.2029 |
|  |  | Triops-Control | -39.11788 | 13.92516 | .141 | -83.7137 | 5.4780 |
|  | Triops-0.75-DMSO | 0.75-2-MNQ | 220.31860^*^ | 13.74547 | .000 | 176.2982 | 264.3390 |
|  |  | 0.75-DMSO | 215.27925^*^ | 13.74547 | .000 | 171.2588 | 259.2997 |
|  |  | 1.5-2-MNQ | 260.33318^*^ | 13.92516 | .000 | 215.7373 | 304.9290 |
|  |  | 1.5-DMSO | 222.51187^*^ | 13.92516 | .000 | 177.9160 | 267.1077 |
|  |  | Control | 213.97197^*^ | 13.92516 | .000 | 169.3761 | 258.5678 |
|  |  | Triops-0.75-2-MNQ | 56.52403^*^ | 13.92516 | .003 | 11.9282 | 101.1199 |
|  |  | Triops-1.5-DMSO | 15.67865 | 13.74547 | .980 | -28.3418 | 59.6991 |
|  |  | Triops-1.5-2-MNQ | 147.13105^*^ | 13.74547 | .000 | 103.1106 | 191.1515 |
|  |  | Triops-Control | 17.40615 | 13.74547 | .960 | -26.6143 | 61.4266 |
|  | Triops-1.5-DMSO | 0.75-2-MNQ | 204.63995^*^ | 13.74547 | .000 | 160.6195 | 248.6604 |
|  |  | 0.75-DMSO | 199.60060^*^ | 13.74547 | .000 | 155.5802 | 243.6210 |
|  |  | 1.5-2-MNQ | 244.65453^*^ | 13.92516 | .000 | 200.0587 | 289.2504 |
|  |  | 1.5-DMSO | 206.83322^*^ | 13.92516 | .000 | 162.2374 | 251.4291 |
|  |  | Control | 198.29332^*^ | 13.92516 | .000 | 153.6975 | 242.8892 |
|  |  | Triops-0.75-2-MNQ | 40.84538 | 13.92516 | .104 | -3.7505 | 85.4412 |
|  |  | Triops-0.75-DMSO | -15.67865 | 13.74547 | .980 | -59.6991 | 28.3418 |
|  |  | Triops-1.5-2-MNQ | 131.45240^*^ | 13.74547 | .000 | 87.4320 | 175.4728 |
|  |  | Triops-Control | 1.72750 | 13.74547 | 1.000 | -42.2929 | 45.7479 |
|  | Triops-1.5-2-MNQ | 0.75-2-MNQ | 73.18755^*^ | 13.74547 | .000 | 29.1671 | 117.2080 |
|  |  | 0.75-DMSO | 68.14820^*^ | 13.74547 | .000 | 24.1278 | 112.1686 |
|  |  | 1.5-2-MNQ | 113.20213^*^ | 13.92516 | .000 | 68.6063 | 157.7980 |
|  |  | 1.5-DMSO | 75.38082^*^ | 13.92516 | .000 | 30.7850 | 119.9767 |
|  |  | Control | 66.84092^*^ | 13.92516 | .000 | 22.2451 | 111.4368 |
|  |  | Triops-0.75-2-MNQ | -90.60702^*^ | 13.92516 | .000 | -135.2029 | -46.0112 |
|  |  | Triops-0.75-DMSO | -147.13105^*^ | 13.74547 | .000 | -191.1515 | -103.1106 |
|  |  | Triops-1.5-DMSO | -131.45240^*^ | 13.74547 | .000 | -175.4728 | -87.4320 |
|  |  | Triops-Control | -129.72490^*^ | 13.74547 | .000 | -173.7453 | -85.7045 |
|  | Triops-Control | 0.75-2-MNQ | 202.91245^*^ | 13.74547 | .000 | 158.8920 | 246.9329 |
|  |  | 0.75-DMSO | 197.87310^*^ | 13.74547 | .000 | 153.8527 | 241.8935 |
|  |  | 1.5-2-MNQ | 242.92703^*^ | 13.92516 | .000 | 198.3312 | 287.5229 |
|  |  | 1.5-DMSO | 205.10572^*^ | 13.92516 | .000 | 160.5099 | 249.7016 |
|  |  | Control | 196.56582^*^ | 13.92516 | .000 | 151.9700 | 241.1617 |
|  |  | Triops-0.75-2-MNQ | 39.11788 | 13.92516 | .141 | -5.4780 | 83.7137 |
|  |  | Triops-0.75-DMSO | -17.40615 | 13.74547 | .960 | -61.4266 | 26.6143 |
|  |  | Triops-1.5-DMSO | -1.72750 | 13.74547 | 1.000 | -45.7479 | 42.2929 |
|  |  | Triops-1.5-2-MNQ | 129.72490^*^ | 13.74547 | .000 | 85.7045 | 173.7453 |
